# A pilot study on the impact of Hydroxiram D supplementation on nutritional status and sarcopenia-related parameters

**DOI:** 10.1007/s13105-026-01253-4

**Published:** 2026-11-12

**Authors:** Paolo Gallo, Valentina Flagiello, Simone Di Cola, Lucrezia Santoro, Dafne Marcelli, Giorgio Reggiardo, Francesca Terracciani, Andrea Falcomatà, Antonio De Vincentis, Antonio Picardi, Raffaele Antonelli-Incalzi, Umberto Vespasiani-Gentilucci

**Affiliations:** 1 Operative Research Unit of Clinical Medicine and Hepatology, Fondazione Policlinico Universitario Campus Bio-Medico, Via Alvaro del Portillo, Roma, 200 - 00128 Italy; 2 Department of Medicine, Karolinska Institutet, H7 Medicin, Huddinge, H7 Endokrinologi och Diabetes Romeo, Stockholm, 171 77 Sweden; 3 Department of Translational and Precision Medicine, Sapienza University of Rome, Rome, Italy; 4 Department of Biostatistics, Consorzio per Valutazioni Biologiche e Farmacologiche (CVBF), Pavia, Italy; 5 Internal Medicine Unit, Fondazione Policlinico Universitario Campus Bio-Medico, Via Alvaro del Portillo, 21, Roma, 00128 Italy; 6 Research Unit of Internal Medicine, Department of Medicine and Surgery, Università Campus Bio-Medico di Roma, Via Alvaro del Portillo, 21, Roma, 00128 Italy; 7 Research Unit of Hepatology, Department of Medicine and Surgery, Università Campus Bio-Medico di Roma, Via Alvaro del Portillo, Roma, 21 - 00128 Italy

**Keywords:** Branched-chain amino acids, Cirrhosis, Hydroxiram D, Beta-hydroxy-beta-methylbutyrate (HMB), Phase angle (PA), Sarcopenia

## Abstract

Sarcopenia is a frequent and prognostically relevant complication of chronic liver disease. The SARCIRAM pilot trial investigated the effect of Hydroxiram D, a formulation of branched-chain amino acids (BCAAs), β-hydroxy-β-methylbutyrate (HMB), and vitamin D, on nutritional status and sarcopenia-related parameters in patients with compensated cirrhosis due to metabolic dysfunction-associated steatotic liver disease (MASLD). In this single-center, randomized pilot study, adults with compensated MASLD-related cirrhosis (Child-Pugh A) received Hydroxiram D plus standard care or standard care alone for 6 months. The primary outcome was phase angle (PA), while secondary outcomes were appendicular skeletal muscle mass (ASMM)/height², handgrip strength (HGS), and 4-meter walk test (4-mWT). Between-group comparisons used baseline-adjusted models. Post-hoc simulation analyses were used exploratorily to inform the design of future studies. Thirty-two patients were analyzed. After 6 months, the adjusted change in PA was significantly more favorable in the treated group versus controls (+ 0.36° vs. − 0.45°, *p* = 0.046). Between-group differences in muscle mass, HGS, and gait speed were not statistically significant, although effect estimates directionally favoured the intervention. No treatment-related adverse events were observed. Six months of Hydroxiram D was associated with a more favorable change in PA in patients with compensated MASLD-related cirrhosis, with favorable trends in muscle parameters. These hypothesis-generating findings warrant confirmation in larger, prospectively registered, double-blind trials to confirm efficacy and long-term outcomes.

## Key points

Hydroxiram D was associated with a more favorable change in phase angle (PA) compared with standard care in MASLD-related cirrhosis. Muscle-related outcomes showed favourable directional trends. Larger placebo-controlled trials are needed.

## Introduction

Sarcopenia is a common complication of chronic liver disease and an independent predictor of liver decompensation, waitlist dropout, and mortality [1–3]. In metabolic dysfunction-associated steatotic liver disease (MASLD)-related cirrhosis, excess adiposity may coexist with progressive impairment of skeletal muscle reserve and may mask early changes in muscle status [4, 5]. Patients with compensated cirrhosis may therefore represent a particularly relevant target for nutritional interventions aimed at preserving muscle mass and function while minimizing the confounding effects of advanced decompensation [6, 7]. The pathogenesis of sarcopenia is multifactorial and involves nutritional deficiency, insulin resistance, systemic inflammation, as well as dysregulation of myostatin signaling, hormonal pathways and hyperammonaemia-driven catabolic signaling [6–9]. These mechanisms contribute to a central and specific metabolic feature of cirrhosis-related sarcopenia, namely accelerated starvation, in which hepatic glycogen depletion promotes the early use of branched-chain amino acids (BCAAs) as a key energy substrate and for ammonia detoxification in skeletal muscle [6, 9]. Nutritional strategies remain the cornerstone of patient management in this context. According to current clinical practice guidelines from the European Association for the Study of the Liver (EASL) and the European Society for Clinical Nutrition and Metabolism (ESPEN), patients with cirrhosis and sarcopenia should achieve a daily protein intake of 1.2–1.5 g/kg and an energy intake of 30–35 kcal/kg [10, 11]. For patients unable to meet these targets through regular meals, oral nutritional supplements, particularly those enriched with BCAAs, are recommended to optimize protein intake and improve nutritional status [10–12], although the supporting evidence is heterogeneous [10–12]. In addition, β-hydroxy-β-methylbutyrate (HMB), a metabolically active derivative of leucine, represents a promising therapeutic target because it stimulates protein synthesis via the mechanistic target of rapamycin (mTOR) and growth hormone/insulin-like growth factor 1 (GH/IGF-1) axis more effectively than leucine alone, while simultaneously inhibiting proteolysis through the ubiquitin-proteasome and autophagy-lysosome systems [13, 14]. Available data derives mainly from early clinical studies focused on hepatic encephalopathy or composite clinical endpoints [15–17]. Finally, vitamin D supplementation is critical, as its deficiency is nearly universal in cirrhosis (64–92%) [18, 19] and correlates with decreased muscle strength, increased insulin resistance, and worse clinical outcomes [19]. However, available evidence supporting a meaningful impact on muscle mass and function remains limited. In this setting, Hydroxiram D appears promising because of its integrated formulation (BCAAs, calcium HMB, and vitamin D) targeting metabolic, anabolic, and endocrine pathways involved in cirrhosis-related muscle impairment (Hydroxiram^®^. Errekappa Euroterapici S.p.A.; 2023. Available upon request). According to this background, the SARCIRAM pilot trial aimed to evaluate the effect of Hydroxiram D on nutritional status (phase angle [PA]) and on sarcopenia-related measures of muscle mass, strength and physical performance in patients with compensated MASLD-related cirrhosis, with particular interest in the potential attenuation of early muscle deterioration.

## Methods

### Study design and population

This was a single-center, open-label, randomized (1:1) pilot study conducted at the Fondazione Policlinico Campus Bio-Medico, Rome, between June 2024 and February 2025. The study protocol conformed to the ethical principles of the Declaration of Helsinki and was approved by the local Ethics Committee (Protocol No. SC 2023.098, approved on 17 May 2023). Although the study protocol was approved before enrolment, this investigator-initiated exploratory pilot trial was not prospectively registered in a public clinical trial registry. We acknowledge this as a methodological limitation of the study. The study was conceived as an exploratory pilot trial aimed at generating preliminary feasibility, safety, and efficacy signals, rather than as a confirmatory trial. Consecutive eligible patients attending routine ultrasonographic surveillance for hepatocellular carcinoma (HCC) were enrolled. Eligibility criteria included age ≥ 18 years, a diagnosis of compensated MASLD-related cirrhosis (Child-Pugh A), no history of liver decompensation according to Baveno VII criteria, and absence of HCC or other active malignancy. Cirrhosis was diagnosed on clinical grounds, based on the presence of specific imaging features of cirrhosis or portal hypertension together with liver stiffness values > 15 kPa [20]. Additional exclusion criteria included presence of any amount of ascites at ultrasound evaluation and/or presence of oedema, acute illness at enrolment and chronic musculoskeletal disorders potentially affecting functional assessments.

### Study intervention and randomization

The study duration was six months. Patients were randomized in a 1:1 ratio to receive either Hydroxiram D (L-leucine 1.25 g, L-isoleucine 0.625 g, L-valine 0.625 g, calcium β-hydroxy-β-methylbutyrate (HMB) 0.75 g, vitamin D 20 µg; Errekappa, Milan, Italy) twice daily in addition to standard care, or standard care alone. The dosing regimen was based on the manufacturer’s recommended posology. All eligible patients received standardized recommendations for diet and usual care prior to randomization. Allocation was performed using computer-generated randomization tables. Treatment adherence was monitored through monthly telephone contacts, during which compliance with the prescribed supplementation and general adherence to dietary recommendations and usual care were qualitatively assessed. Habitual dietary intake, including daily energy and protein intake, was not prospectively quantified using standardized food diaries, dietary recalls or validated dietary questionnaires.

### Baseline and outcome assessment

Demographic data, anthropometric parameters, liver function tests, and nutritional parameters were collected for each participant at baseline (T0) and after six months of follow-up (T1). Muscle mass was evaluated by bioelectrical impedance analysis (BIA), muscle strength by handgrip strength (HGS), and physical performance by the 4-meter walk test (4-mWT), as recommended and defined by the European Working Group on Sarcopenia in Older People 2 (EWGSOP2) [21]. The primary outcome was the change in phase angle (PA) from baseline to 6 months. Secondary outcomes included changes in appendicular skeletal muscle mass (ASMM) indexed to height squared (ASMM/h²), handgrip strength (HGS), and 4-mWT.

### Bioelectrical impedance analysis

Whole-body BIA was performed by a trained physician using the BIA 101 BIVA PRO device (Akern, Florence, Italy), which delivers a constant current at 50 kHz. Participants fasted for at least six hours and were examined in the supine position, with slightly abducted limbs, after a resting period of at least two minutes. Measurements lasted less than 20 s and were obtained using a tetrapolar configuration to assess resistance (Rz) and reactance (Xc). From single-frequency whole-body BIA, total body water (TBW, kg and %), fat mass (FM, kg and %), fat-free mass (FFM, kg and %), and PA, ° were derived. ASMM was estimated using the equation proposed by Sergi et al. [22] and normalized for height (ASMM/h² or BIA-Skeletal Muscle Index [SMI]). Low muscle mass was defined according to EWGSOP2 criteria (ASMM/h² <7.0 kg/m² for men and < 5.5 kg/m² for women) [21]. All BIA-derived parameters were collected at T0 and T1. BIA was considered appropriate in this compensated cirrhotic cohort (Child-Pugh A), as participants were clinically free of significant ascites or fluid overload, minimizing potential bias related to extracellular water expansion.

### Handgrip strength

Muscle strength was assessed by HGS using a Jamar dynamometer. Measurements were performed with the participant seated and the elbow flexed at 90°. Three maximal voluntary contractions were recorded for each hand, and the highest value obtained across all six measurements, irrespective of hand dominance, was used for analysis. Dynapenia was defined according to EWGSOP2 cut-off values (HGS < 27 kg for men and < 16 kg for women) [21].

### Four-meter walk test

Physical performance was evaluated using the 4-mWT. Participants were instructed to walk at their habitual pace along a straight, marked 4-meter corridor. Timing started at the first foot movement and stopped when the leading foot crossed the finish line. The test was performed twice, and the mean time required to complete 4 m was recorded. Gait speed (m/s), calculated as 4 m divided by the time required to complete the test, was used for analysis. According to EWGSOP2 criteria, impaired performance corresponds to a gait speed < 0.8 m/s, equivalent to a time > 5 s to complete 4 m [21].

### Statistical analysis

All collected data were recorded in an electronic medical record (EMR). No formal a priori power calculation was performed. Given the exploratory pilot nature of the study, a pragmatic target sample of 40 participants (20 per group) was selected, informed by previous pilot studies and intended to provide preliminary estimates of feasibility, variability and treatment effect for the design of future confirmatory trials [17]. The efficacy analysis was conducted in a modified evaluable analysis set comprising randomized participants with available baseline and 6-month outcome data. Analyses of individual outcomes were conducted on available cases with both baseline and 6-month measurements; therefore, the number of participants included in individual analyses could vary according to outcome. Missing outcome data were not imputed. Participants without evaluable follow-up outcomes or found after randomization to meet exclusion criteria were not included in the efficacy analysis. Continuous variables are reported as mean ± standard deviation (SD) or median and interquartile range (IQR), as appropriate, while categorical variables are expressed as frequencies and percentages. Between-group comparisons at each individual visit were performed using one-way analysis of variance (ANOVA) for continuous variables and Fisher’s exact test for categorical variables. Between-group differences in changes from baseline to 6 months were assessed using analysis of covariance (ANCOVA), with treatment group as the main factor and baseline body mass index (BMI), baseline TBW, and baseline value of the corresponding outcome as covariates. Results are presented as adjusted means (estimated marginal means, EMMs). Within-group changes over time were evaluated using paired t-tests or Wilcoxon signed-rank tests, depending on data distribution. Normality was assessed using the Shapiro-Wilk test. A 2-sided p-value < 0.05 was considered statistically significant. A post-hoc power analysis based on a modelling and simulation approach was conducted to explore power variability and to inform effect-size and sample-size considerations for future confirmatory studies.

## Results

### Baseline characteristics of the study population

**Fig. 1:**
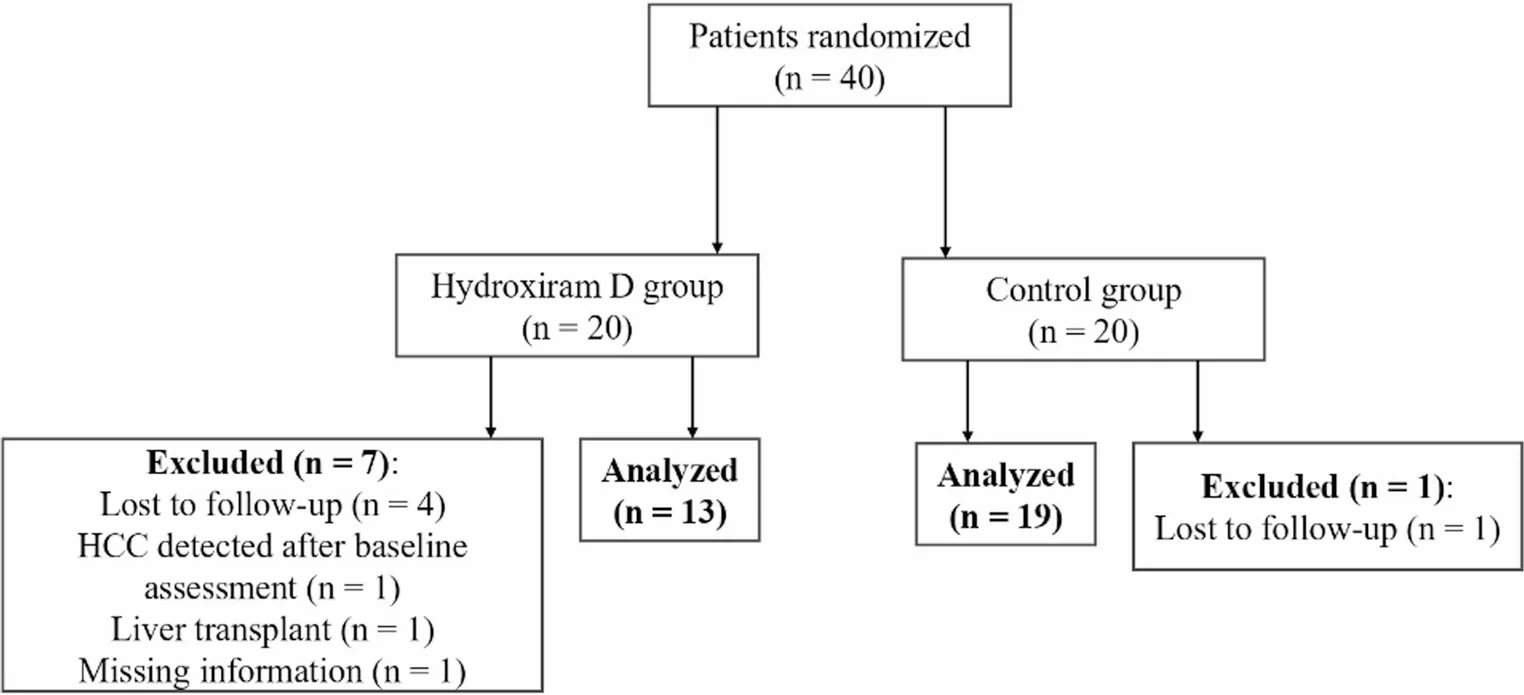
Study flow diagram. A total of 40 patients were randomized and allocated to the Hydroxiram D group (*n* = 20) or the control group (*n* = 20). After exclusions, 32 patients completed the study and were included in the final analysis (Hydroxiram D group, *n* = 13; control group, *n* = 19). *HCC*, hepatocellular carcinoma

Among the 40 randomized patients, 32 completed the study (Hydroxiram D, *n* = 13; control, *n* = 19) (Fig. 1). During the 6-month follow-up, no episodes of hepatic decompensation, including ascites, hepatic encephalopathy or variceal bleeding, occurred in either treatment group. In the Hydroxiram D group, two participants developed HCC; one was excluded following HCC diagnosis, whereas the other underwent liver transplantation for HCC. The transplantation was not prompted by hepatic decompensation.

The completion rate was lower in the Hydroxiram D arm (65%, 13/20) than in the control group (95%, 19/20). Discontinuations occurred due to loss to follow-up (4), HCC detection after baseline assessment (1), liver transplantation following HCC detection during follow-up (1), and missing information (1). In the control group, one participant was lost to follow-up (1). Baseline demographic, clinical, and nutritional characteristics were comparable between groups. Mean age was 71.1 ± 8 years in the Hydroxiram D group and 72.5 ± 7.8 years in controls, with a higher proportion of women in the Hydroxiram D arm (69.2% vs. 47.4%). BMI was similar across treatment arms (30.5 ± 5.3 kg/m² in Hydroxiram D group and 29.6 ± 4.2 kg/m² in the control group). Routine laboratory parameters, including liver function tests, renal function, nutritional markers, and serum 25-hydroxyvitamin D levels, were well balanced between groups (Table 1). At baseline, no participant reported regular physical activity. Baseline body composition and muscle-related parameters did not differ between arms. At study entry, the prevalence of low muscle mass, defined according to EWGSOP2 criteria, was low in both groups (Hydroxiram D: 1/13, 7.7%; control: 3/19, 15.8%). FFM, PA, and ASMM/h² were also comparable between groups. Similarly, muscle strength assessed by HGS and physical performance measured by 4-mWT showed no relevant between-group differences at study entry (Table 1).

**Table 1:** Baseline and follow-up clinical, biochemical, and body composition characteristics according to treatment group

	Visit 0 (baseline)			Visit 1 (follow-up)		
	Hydroxiram D	Control	*p*-value	Hydroxiram D	Control	*p*-value
Age, years	71.1 ± 7.97	72.5 ± 7.76	–	–	–	–
Women, n (%)	9 (69.2%)	9 (47.4%)	–	9 (69.2%)	9 (47.4%)	–
Men, n (%)	4 (30.8%)	10 (52.6%)	–	4 (30.8%)	10 (52.6%)	–
BMI, kg/m²	30.45 ± 5.26	29.58 ± 4.16	0.63	29.86 ± 5.43	29.47 ± 4.05	0.82
AST, U/L	29.34 ± 8.22	32.94 ± 10.17	0.315	29.75 ± 6.36	38.37 ± 16.61	0.098
Albumin, g/dL	4.10 ± 0.40	4.11 ± 0.42	0.984	4.12 ± 0.28	3.90 ± 0.38	0.106
Creatinine, mg/dL	1.04 ± 0.54	1.06 ± 0.41	0.920	0.95 ± 0.43	0.98 ± 0.32	0.802
Urea, mg/dL	49.73 ± 24.73	46.9 ± 19.36	0.750	46.11 ± 16.59	43.12 ± 13.59	0.601
Vitamin D, ng/mL	27.32 ± 10.40	23.91 ± 9.13	0.382	33.37 ± 5.41	24.74 ± 13.20	0.061
Ammonia, µmol/L	81.45 ± 107.84	64.70 ± 20.83	0.742	50.39 ± 22.03	43.23 ± 23.97	0.432
FFM, kg	49.7 ± 11.76	53.1 ± 11.45	0.411	47.7 ± 11.26	50.5 ± 10.29	0.472
FFM, %	65.4 ± 6.74	67.7 ± 7.83	0.401	62.9 ± 6.10	65.3 ± 8.15	0.384
PA, °	5.32 ± 0.93	5.26 ± 1.62	0.905	5.49 ± 1.64	4.94 ± 0.92	0.234
ASMM/h², kg/m²	6.99 ± 0.96	7.50 ± 1.36	0.249	6.76 ± 1.02	7.03 ± 1.15	0.498
HGS, kg	24.54 ± 13.60	23.89 ± 12.27	0.890	26.92 ± 14.52	25.68 ± 13.79	0.809
4-mWT, m/s	0.917 ± 0.47	1.167 ± 0.49	0.173	0.841 ± 0.32	0.906 ± 0.29	0.563

Data are expressed as mean ± SD or n (%), as appropriate. P-​values refer to between-group comparisons at each visit (Visit 0 and Visit 1) and were calculated using one-way ANOVA for continuous variables and Fisher’s exact test for categorical variables. *ANOVA*, analysis of variance; *ASMM/h²*, appendicular skeletal muscle mass indexed to height squared; *AST*, aspartate aminotransferase; *BMI*, body mass index; *FFM*, fat-free mass; *HGS*, handgrip strength; *PA*, phase angle; *SD*, standard deviation; *4-mWT*, four-meter walk test

### Treatment effects at 6 months

**Fig. 2:**
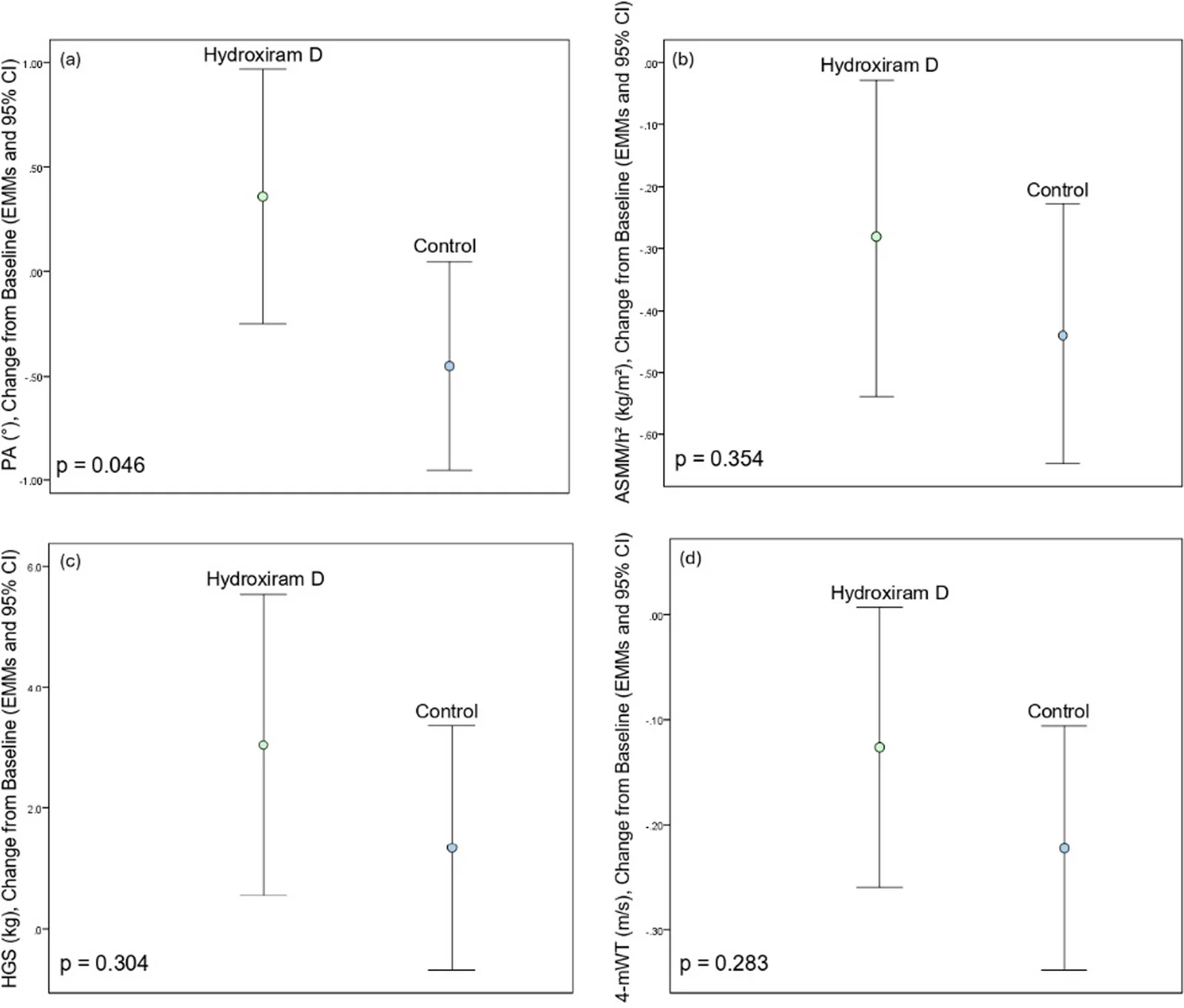
ANCOVA-adjusted changes in muscle mass and physical function outcomes according to treatment group. Panels show estimated marginal means (EMMs) of change from baseline to end of study with 95% CI derived from ANCOVA models adjusted for baseline BMI, TBW, and baseline value of each outcome: (**a**) PA, (**b**) ASMM/h², (**c**) HGS, and (**d**) 4-mWT. P-values refer to between-group comparisons. *ANCOVA*, analysis of covariance;* ASMM/h²*, appendicular skeletal muscle mass indexed to height squared; *BMI*, body mass index; *CI*, confidence interval; *EMMs*, estimated marginal means; *HGS*, handgrip strength; *PA*, phase angle; *TBW*, total body water; *4-mWT*, four-meter walk test

Treatment effects were evaluated using ANCOVA models adjusted for baseline BMI, TBW, and the baseline value of each outcome. After 6 months of follow-up, a significantly more favourable change in PA was observed in the Hydroxiram D group compared with controls. The adjusted mean change in PA was + 0.36° (95% CI − 0.25 to + 0.97) in the treated group versus − 0.45° (95% CI − 0.95 to + 0.05) in the control group, reaching statistical significance (*p* = 0.046) (Fig. 2; Table 2).

**Table 2:** ANCOVA-adjusted changes from baseline in body composition and physical function outcomes according to treatment group

	Hydroxiram D group	Control group	ANCOVA *p*-value
FFM, kg	−1.89 (− 3.21 to − 0.57)	−2.69 (− 4.10 to − 1.28)	0.431
PA, °	+ 0.36 (− 0.25 to + 0.97)	−0.45 (− 0.95 to + 0.05)	0.046
ASMM/h², kg/m²	−0.28 (− 0.54 to − 0.03)	−0.44 (− 0.65 to − 0.23)	0.354
HGS, kg	+ 3.04 (+ 0.56 to + 5.53)	+ 1.34 (− 0.69 to + 3.37)	0.304
4-mWT, m/s	−0.126 (− 0.245 to − 0.007)	−0.222 (− 0.349 to − 0.095)	0.283

Data are expressed as estimated marginal means (EMMs) with 95% confidence intervals. P-values refer to between-group comparisons and were derived from ANCOVA models. *ANCOVA*, analysis of covariance; *ASMM/h²*, appendicular skeletal muscle mass indexed to height squared; *BMI*, body mass index; *FFM*, fat-free mass; *HGS*, handgrip strength; *PA*, phase angle; *4-mWT*, four-meter walk test

**Fig. 3:**
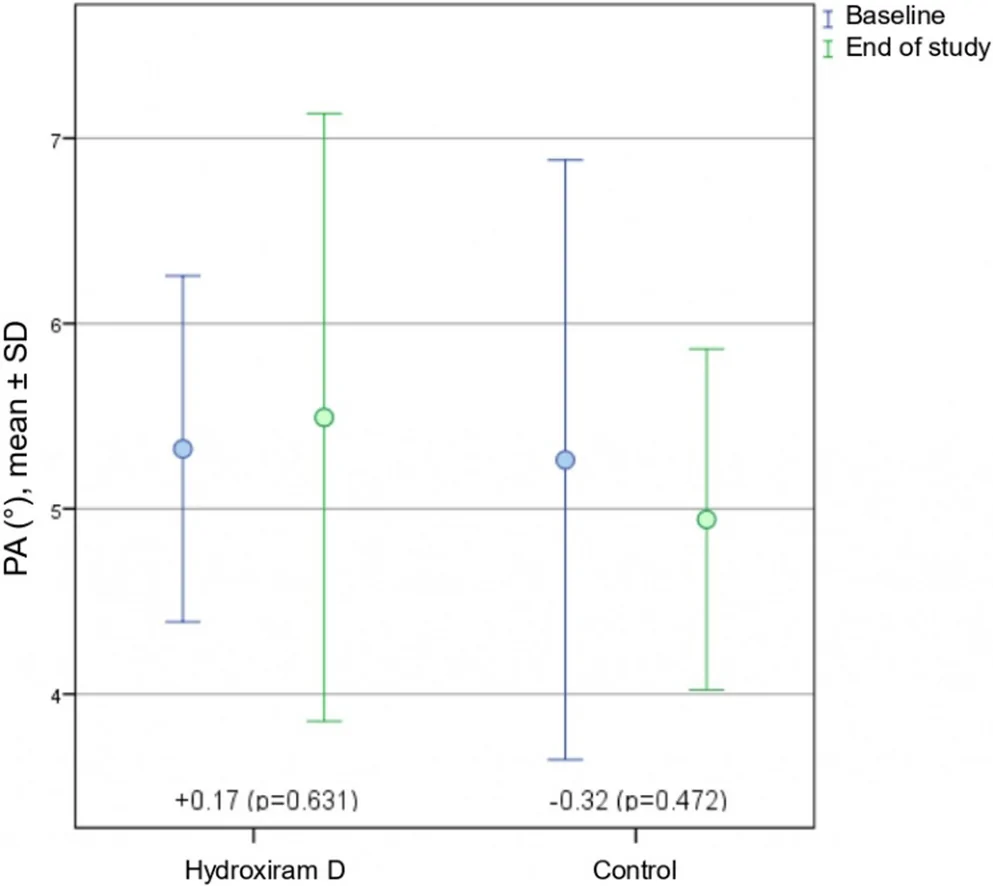
Phase Angle at baseline and end of study according to treatment group. Mean ± standard deviation of PA at baseline and at end of study in the Hydroxiram D and control groups. Blue symbols represent baseline values and green symbols represent end-of-study values. Within-group changes from baseline and corresponding p-values are shown for exploratory purposes. *PA*, phase angle

ASMM/h² declined in both groups over the study period. The adjusted mean decrease was smaller in the Hydroxiram D group compared with controls (− 0.28 vs. − 0.44 kg/m²), although the between-group difference did not reach statistical significance (*p* = 0.354) (Table 2). Similarly, no significant between-group differences were observed for muscle strength or physical performance. ANCOVA-adjusted changes in HGS were + 3.04 kg in the Hydroxiram D group and + 1.34 kg in controls (*p* = 0.304), while 4-mWT declined by − 0.126 m/s and − 0.222 m/s, respectively (*p* = 0.283) (Table 2). Despite the lack of statistical significance, the direction of change across these functional outcomes consistently favoured the treated group. In line with previous findings, within-group analyses (Fig. 3) showed that PA increased over time in the Hydroxiram D group, declining in the control group, and similar trends were observed for ASMM/h² and physical performance measures, supporting the consistency of the between-group findings. Finally, no significant between-group differences were observed for TBW variations.

No treatment-related adverse events were reported, and routine laboratory parameters remained clinically stable throughout the study period. Importantly, no discontinuation was attributed to intolerance or adverse events.

### Post-hoc power considerations

A post-hoc, simulation-based power analysis was conducted to contextualize the magnitude of the effect sizes observed in this pilot study. Specifically, simulated adjusted changes suggested differences between treated patients and controls for PA (+ 0.179° vs. − 0.357°, *p* < 0.001), ASMM/h² (–0.289 vs. − 0.443 kg/m²; *p* < 0.001), and 4-mWT (–0.140 vs. − 0.210 m/s; *p* < 0.001), respectively (Table 3). These exploratory analyses support the consistency of the observed effect for PA and the directional trends for ASMM/h^2^ and functional outcomes, which may be further evaluated in larger, adequately powered studies.

**Table 3:** Simulated (in post-hoc analysis) ANCOVA-adjusted changes from baseline in body composition and physical function outcomes according to treatment group

	Hydroxiram D group	Control group	Simulated ANCOVA *p*-value
PA, °	+ 0.179 (0.016 to 0.343)	−0.357 (− 0.492 to − 0.222)	< 0.001
ASMM/h², kg/m²	−0.289 (− 0.331 to − 0.247)	−0.443 (− 0.497 to − 0.389)	< 0.001
HGS, kg	+ 2.340 (1.754 to 2.926)	+ 1.827 (1.344 to 2.309)	0.185
4-mWT, m/s	−0.140 (− 0.162 to − 0.118)	−0.210 (− 0.229 to − 0.191)	< 0.001

Data are expressed as ANCOVA-adjusted mean changes from baseline (T1–T0) with 95% CI. Values derive from a post-hoc simulation-based analysis using ANCOVA models fitted on the full analysis set and expanded to a synthetic cohort (*n* = 500) mirroring the observed covariate structure and variance. Models were adjusted for baseline value of the outcome, body mass index, and TBW. Results are exploratory and intended to contextualize effect size magnitude rather than to provide confirmatory evidence. P-values refer to simulated between-group comparisons. *ANCOVA*, analysis of covariance; *ASMM/h²*, appendicular skeletal muscle mass indexed to height squared; *CI*, confidence interval; *HGS*, handgrip strength; *PA*, phase angle; *TBW*, total body water; *4-mWT*, four-meter walk test

## Discussion

In this small pilot randomized study, supplementation with Hydroxiram D was associated with a significantly more favourable change in PA over six months in patients with compensated MASLD-related cirrhosis, compared with standard care alone. Although no statistically significant between-group differences were observed for ASMM/h^2^, HGS, or physical performance, effect estimates were directionally consistent and uniformly favoured the treated group.

PA is increasingly recognized as an integrative bioelectrical marker reflecting nutritional status, body cell mass and cell membrane integrity rather than a direct measure of muscle quantity [23, 24]. In patients with cirrhosis, lower PA values have been associated with frailty, hospitalization and mortality, supporting its prognostic relevance [25–28]. In the present study, the adjusted mean change in PA was + 0.36° in the Hydroxiram D group and − 0.45° in controls, corresponding to an adjusted between-group difference of approximately 0.8°. However, a validated minimal clinically important difference for PA has not been established in patients with compensated cirrhosis. Therefore, the magnitude of the observed difference cannot be directly translated into a reduction in clinically relevant outcomes. Rather, it should be interpreted as a potentially relevant nutritional and bioelectrical signal that requires confirmation against clinical outcomes in adequately powered longitudinal studies. The absence of a significant between-group change in total body water reduces, although does not exclude, the possibility that differences in hydration contributed to the observed PA change.

Although ASMM/h^2^ declined in both groups, the magnitude of decline was numerically smaller in treated patients. Similarly, changes in HGS and physical performance did not differ significantly between groups after adjustment but showed a consistent trend in favour of the Hydroxiram D group.

The biological basis of the observed PA difference is likely multifactorial. PA is determined by the relationship between tissue resistance and reactance and indirectly reflects the capacitive properties of cell membranes, body cell mass and fluid distribution [6, 24, 25]. Leucine-rich BCAAs may support amino-acid availability and mTOR complex 1 (mTORC1)-mediated anabolic signalling, whereas HMB may attenuate proteolytic pathways and favour protein turnover; vitamin D may additionally contribute to muscle metabolic and contractile function. However, these pathways were not directly investigated in the present study, and mechanistic interpretations therefore remain speculative [13, 16, 19].

Our findings can be placed in the context of previous nutritional intervention studies in cirrhosis. A recent randomized pilot trial in patients with stable decompensated cirrhosis and established sarcopenia reported an increase in PA from 4.6° to 5.0° after 12 weeks in participants receiving an HMB-containing oral nutritional supplement, although no significant between-group differences were observed in muscle mass or handgrip strength [29]. Conversely, Lattanzi et al. did not observe a significant change in PA during 12 weeks of HMB supplementation in a predominantly compensated cirrhotic population, despite improvements in selected ultrasound-derived and functional measures [17]. Differences in disease severity, baseline nutritional and sarcopenia status, supplement composition and follow-up duration may contribute to these heterogeneous findings. Thus, the present PA result is biologically plausible but should be considered preliminary rather than evidence of established clinical efficacy.

An important consideration is that low muscle mass was uncommon at baseline and that the study population predominantly consisted of patients without overt sarcopenic features.

This study has several limitations, including its small sample size, open-label nature, single-center design and relatively short duration. Therefore, it should be considered hypothesis-generating. In particular, the 6-month follow-up may have been insufficient to detect meaningful changes in skeletal muscle mass, physical performance, or incident sarcopenia, especially in clinically stable patients with compensated cirrhosis, in whom muscle deterioration may progress relatively slowly. Accordingly, the study may have been more sensitive to early nutritional or bioelectrical changes than to definitive structural or functional changes. In addition, while no discontinuations were related to intolerance or adverse events, the imbalance in the final analyzed sample due to losses to follow-up and post-randomization exclusions may have introduced attrition bias, reduced the balance originally achieved through randomization, and limited the internal validity and precision of efficacy estimates in this small pilot trial. Furthermore, habitual dietary intake was not prospectively quantified using standardized dietary assessment tools; therefore, individual energy and protein intake during follow-up was unavailable. Although both groups received the same dietary recommendations, unmeasured dietary variation may have influenced nutritional and muscle-related outcomes, and the specific contribution of Hydroxiram D cannot be completely disentangled from these potential co-interventions. In addition, although BIA was considered suitable for serial assessment in this clinically compensated population without ascites or peripheral oedema, computed tomography (CT)-derived skeletal muscle quantification at the third lumbar vertebral (L3) level remains the best-validated reference approach for muscle mass assessment in cirrhosis. The absence of systematic CT-based muscle measurements therefore limits direct comparison with established imaging-based definitions of sarcopenia. In addition, the complete components required to calculate a validated liver frailty index were not systematically collected, preventing retrospective assessment of longitudinal changes in frailty. Nevertheless, the observed between-group difference in PA, and the consistency of directional trends across secondary outcomes, provide a preliminary signal supporting further evaluation of this intervention. Post-hoc modelling and simulation analyses may provide useful exploratory information for planning future studies but should not be interpreted as increasing the evidentiary strength of the findings observed in the original pilot sample or as confirmatory evidence of treatment efficacy. Moreover, the lack of prospective registration in a public clinical trial registry represents an additional limitation. The study protocol was approved by the local Ethics Committee before enrolment and all participants provided written informed consent; nevertheless, the absence of prospective registration reduces the external verifiability of prespecified outcomes and analyses and should be considered when interpreting these exploratory findings. Future confirmatory trials should be prospectively registered, adequately powered, double-blind, placebo-controlled and include longer follow-up, standardized assessment of dietary intake, imaging-based muscle phenotyping, and longitudinal frailty and clinical outcomes in order to confirm these findings and clarify their clinical implications.

## Conclusion

In patients with compensated MASLD-related cirrhosis, six-month supplementation with a combined formulation of BCAAs, HMB, and vitamin D was associated with a significantly favourable change in PA, an integrative marker of nutritional status and cellular integrity, compared with standard care. Although no significant between-group effects on muscle mass, muscle strength or physical performance were observed, consistent directional trends favoured the treated group. These findings warrant confirmation in larger, adequately powered trials.

## Data Availability

The data presented in this study are available on reasonable request from the corresponding author. The data are not publicly available due to privacy and ethical restrictions related to individual clinical data.
